# Risk of predation makes foragers less choosy about their food

**DOI:** 10.1371/journal.pone.0187167

**Published:** 2017-11-09

**Authors:** Alice Charalabidis, François-Xavier Dechaume-Moncharmont, Sandrine Petit, David A. Bohan

**Affiliations:** 1 Agroécologie, AgroSup Dijon, INRA, Université de Bourgogne Franche-Comté, Dijon, France; 2 Université de Bourgogne Franche-Comté, UMR CNRS 6282 Biogéosciences, Evolutionary Ecology group, Dijon, France; University of Vienna, AUSTRIA

## Abstract

Animals foraging in the wild have to balance speed of decision making and accuracy of assessment of a food item’s quality. If resource quality is important for maximizing fitness, then the duration of decision making may be in conflict with other crucial and time consuming tasks, such as anti-predator behaviours or competition monitoring. Individuals facing the risk of predation and/or competition should adjust the duration of decision making and, as a consequence, their level of choosiness for resources. When exposed to predation, the forager could either maintain its level of choosiness for food items but accept a reduction in the amount of food items consumed or it could reduce its level of choosiness and accept all prey items encountered. Under competition risk, individuals are expected to reduce their level of choosiness as slow decision making exposes individuals to a higher risk of opportunity costs. To test these predictions, the level of choosiness of a seed-eating carabid beetle, *Harpalus affinis*, was examined under 4 different experimental conditions of risk: i) predation risk; ii) intraspecific competition; iii) interspecific competition; and, iv) control. All the risks were simulated using chemical cues from individual conspecifics or beetles of different species that are predatory or granivorous. Our results show that when foraging under the risk of predation, *H*. *affinis* individuals significantly reduce their level of choosiness for seeds. Reductions in level of choosiness for food items might serve as a sensible strategy to reduce both the total duration of a foraging task and the cognitive load of the food quality assessment. No significant differences were observed when individuals were exposed to competition cues. Competition, (i.e opportunity cost) may not be perceived as risk high enough to induce changes in the level of choosiness. Our results suggest that considering the amount of items consumed, alone, would be a misleading metric when assessing individual response to a risk of predation. Foraging studies should therefore also take in account the decision making process.

## Introduction

Mating or feeding enough to maintain fitness is a significant challenge in a world where resources can vary markedly in availability and quality. When sampling resources, individuals encounter items that do not fulfil their needs [1] or that are hazardous, either by being poisonous (e.g. stinging insects [2]) or by harbouring predators, such as crab spiders camouflaged in flowers attractive to insect pollinators [3,4]. Thus, the fitness of an individual would increase with its ability to accurately evaluate the quality of a resource, and decide between accepting an item immediately available or waiting for a potentially better future option, but with no guarantees as to the outcome. Such precise evaluation, however, gives rise to incompressible cognitive and time costs. An individual seeking resources should therefore experience a speed accuracy trade-off while choosing which item to exploit [5,6].

Investing too much time in assessing the quality of a resource item could be detrimental for individuals foraging or seeking a mate under hazardous situations, such as risks of predation or competition [7–10]. Under the risk of predation, individuals deal with two conflicting tasks [4,11–14] or mutually exclusive behaviours [15,16]: either the avoidance of predators or the acquisition of resources (a vigilance-foraging trade-off). Given the immediate and lethal outcome of failing to avoid a predator, a potential prey individual should adjust its foraging behaviour primarily to the predation risk and only secondarily to starvation [15]. Thus, individuals are expected to postpone foraging tasks and allocate more time and energy to predator avoidance behaviours, when under no energy stress [17].

Postponement of foraging is only a sensible strategy for short periods of predation risk, however, it could be hazardous during extended periods of diffuse predation risk or when the risk of starvation is too high to defer foraging [17]. An animal is expected to adjust its foraging effort in respect of its energetic requirements and the likelihood of predator attack [18,19]. It supposes that the individuals are able to, firstly, assess local predation risk and, secondly, adjust their intensity of an antipredator response according to the level of threat [20–23]. When assessing the response to a predation risk during foraging, the authors typically measured the number of food items consumed per unit of time, which is based on the assumption of direct proportionality between the number of items consumed and the “feeding effort” (i.e. the time spent foraging). Under such an assumption, the predicted decrease in the time spent foraging under predation risk would result in an overall decrease of the number of food items consumed [7,24]. A rarely considered alternative assumption, which we consider in this study, is that an individual adjusts its foraging strategy while keeping constant the number of food items consumed. To mitigate a vigilance-foraging trade off, the forager might adjust the time spent in assessing a resource item before deciding whether or not to accept it, rather than simply reducing the amount of food collected [25,26].

The time or energy that an individual invests in sampling or assessing an available resource item is termed ‘choosiness’ in the behavioral literature [10,27]. Choosy individuals accept only a few resources in a given time span or spend a substantial amount of time assessing an item before accepting it, whereas less choosy individuals either accept more resource items over the same time or hesitate for a shorter amount of time before consuming a resource item [27,28]. Consequently, the time spent in assessment before accepting an encountered resource item is a primary metric for evaluating individual choosiness. When exposed to predation risk, a forager should increase the time allocated to anti-predator behaviours and, thus, reduce the total time invested in foraging [18]. This could result in two apparently contradictory foraging patterns: i) a forager could reduce the length of the foraging period, while maintaining a constant level of choosiness, leading to an observed reduction of the number of food items consumed [7,24,29]; or, ii) an individual could reduce its level of choosiness, by accepting all prey items encountered irrespective of their quality [8,30], and keep constant the number of food items consumed [31]. This last pattern might erroneously be interpreted as an absence of behavioural flexibility in response to predation risk, if the number of prey eaten were recorded alone. The total amount of items consumed should not serve as the sole metric for assessing the behavioural adjustment to risks. More specifically, studies that have found no adjustment of foraging effort in response to predation risk [19], may have done so because they considered only the total amount of items consumed and neglected the variation in individual choosiness under predation risk.

Adjustment of choosiness may also be an important behavioural response to competition [32–35]. In the absence of competition, one sensible strategy would be to select and consume only the most profitable food resources, and neglect most of the encountered items. Where competitors are also present in the same patch, however, such a choosy forager might be unable to fulfil its energetic needs. Neglecting food items of low quality, in this way, is costly because the expected better items could have already been consumed by competitors. Moreover, choosy foragers may not be able to re-adjust their thresholds of prey acceptability, following a lengthy unsuccessful period, because lower choice items that had been previously neglected might have already been consumed by less choosy competitors [10]. These lost opportunity costs can be sufficiently strong to constrain the evolutionary stable strategy for prey choosiness. Indeed, game-theoretical approaches suggest that optimal level of choosiness is frequency-dependent and decreases with increasing competition [10].

Our hypothesis is that both predation and competition risks affect levels of choosiness. As the fitness costs of predation should be higher and more immediate than the costs resulting from competition, differences in the intensity of either an increase or reduction of the level of choosiness (behavioural adjustment) under each of these two risks is expected. We examine whether individuals of a granivorous carabid species, *Harpalus affinis* (Schrank, 1781), modulate their level of choosiness for seeds as a function of either predation risk or competition, from either intraspecific or interspecific competitors.

## Methods

### Study system

The carabid species used in the tests are commonly found together in European farmland. *H*. *affinis* is a granivorous species that we use as our focal test forager as it is one of the most abundant spring-breeding predominantly granivorous species in arable agriculture. We chose to use *Pterostichus melanarius* (Illiger, 1798) as the potential predator because they have been shown to be voracious predators of live prey [36–40]. Moreover, *P*. *melanarius* were observed to prey upon *H*. *affinis* in experimental situations (Alice Charalabidis, pers. obs.), and upon others species of carabids [40]. *Pseudoophonus rufipes* (De Geer, 1774) was chosen as the interspecific competitor, given that this granivorous species has been observed to readily eat a large amounts of seeds in laboratory conditions [41]. We sampled adult individuals of three carabids species, *H*. *affinis*, *P*. *melanarius* and *P*. *rufipes* on the INRA experimental farm at Epoisses (Côte d’Or, France; 47°14’11.4”N 05°05’53.4”E) using pitfall traps during spring and summer 2015. Individuals of the focal species, *H*. *affinis*, were maintained in small, mixed sex groups (up to 20 individuals) in plastic boxes (34 x 19 x 11 cm, length x breadth x height) for a minimum of two weeks prior to experimentation. Each box contained two to three cm deep soil and some moistened paper tissue to maintain high humidity and provide the carabids with shelter. The boxes were maintained under temperature- and light-controlled conditions (19°C +/- 1°C, 60% humidity, 14:10h light:dark cycle). Boxes of *H*. *affinis*, the granivore *P*. *rufipes* and the omnivore *P*. *melanarius* were kept in separate rooms to prevent interspecific predation [40] or any possible effects of chemical cues. Age, mated status and feeding background were not controlled as we used wild-caught individuals in the tests.

Highly preferred seeds might induce high risk taking by the carabids, and therefore acceptance in all contexts of risk, while disliked seeds would not be accepted at any level of risk. *T*. *officinale*, a moderately preferred species [41,42] that is known to be eaten both by *H*. *affinis* and *P*. *rufipes*, was therefore selected as the test seed. In order to standardize their feeding background and ensure that *T*. *officinale* seeds were encountered at least once by all tested individuals prior to the experiment [43], individuals of *H*. *affinis* were fed with a combination of four seeds species, *T*. *officinale*, *Viola arvensis* (Murray), *Senecio vulgaris* L. and *Capsella bursa-pastoris* (L.) Medik. All seeds were one year old and were collected on the INRA Dijon experimental farm. All experimental seeds had been soaked in water for 14 hours to become more palatable to and detectable by carabids [44]. Carabids were provided with water *ad libitum* in an Eppendorf containing moistened cotton wool.

### Experimental set-up

The 290 experimental *H*. *affinis* individuals were randomly split into four treatment groups, *control* (n = 70, with 31 females and 39 males), *intraspecific competition (*n = 71, with 32 females and 39 males), *interspecific competition* (n = 75, with 32 females and 43 males), and *predation* (n = 74, with 31 females and 43 males). The sexes were identified using protarsi, which are dilated and have hairy undersides in males [45]. All beetles were tested individually and only once. To standardize the feeding motivation, individuals were isolated in small individual plastic boxes (diameter 9 cm) and starved for the 54 hours prior to testing. Starvation duration was estimated from pre-test experiments designed to produce individuals motivated to feed, but not so starved that they were too tired to forage. Water was provided *ad libitum* via a moist paper tissue covering the bottom of each individual boxes.

**Fig 1:**
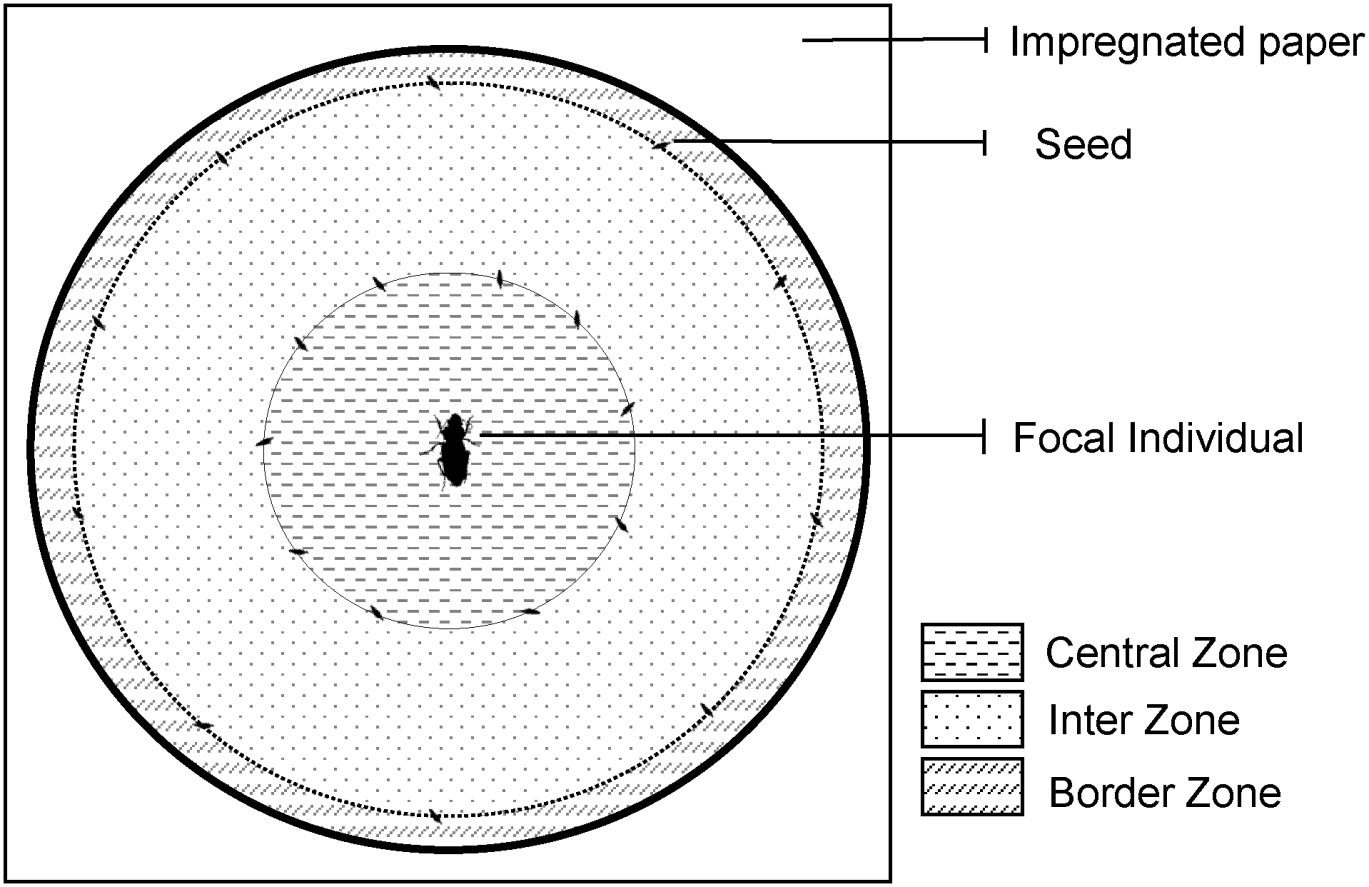
Design of the test arena. Arena was divided intro into three circular parts by two circles of respectively 5 cm and 16 cm diameter: i) the central zone, ii) the inter-zone and iii) the border zone. Ten *T*. *officinale* seeds were placed around each circle. This representation is approximately to scale: carabids measure ~1 cm and seed ~2.5 mm in length.

Predation and competition risk were simulated via olfactory cues that carabids leave along their path of movement [46,47]. Using these cues, in place of live predators or competitors, we avoided the confounding effects of direct interactions between the focal individual and predators and competitors. The consistency of the chemicals cues was tested by Guy et al. [46], who found that carabids responded to almost 2 day old residual chemicals. To simulate the risk of predation, we used the chemicals cues left by *P*. *melanarius*. Interspecific competition was simulated using chemicals from *P*. *rufipes* and intraspecific competition was simulated using chemicals from *H*. *affinis*. Using the method of Armsworth *et al*. [48], impregnated papers (white filter paper, Dutscher, Brumath, France) were created by allowing 20 individual beetles (10 females, 10 males) to walk over test papers (40 x 30 cm) for a minimum of 24 hours; this density of stimulus individuals has been previously shown to induce concentration of olfactory cues which is perceived by carabids [46]. For the control treatment clean test papers, with no carabid chemical cues, were used. We used two different types of competition in order to differentiate potential sexual induced-behaviours in the intraspecific competition treatment from actual behavioural responses to the cues of competition risk. The impregnated test papers were collected immediately prior to the start of each experimental trial. For each experimental arena we arranged 20 seeds of *T*. *officinale* in two concentric circles of 5 and 16 cm diameter on an impregnated test paper (Fig 1).

The focal carabid individual was acclimatized under a plastic pot at the centre of the arena for 8 minutes. The pot was removed and we immediately placed an inverted 18 cm diameter Pyrex petri dish bottom over the arena to delimit and isolate the arena from external perturbations (movement of air, chemical cues). Foraging behaviours were then scored over a one hour period. The test papers were used for only one trial, and between repetitions the petri dishes were washed in a medical dish-washer.

The four experimental conditions and the two sexes of *H*. *affinis* were tested in random order in controlled temperature room at 19°C +/- 1°C and 60% humidity. The arenas were laid out on an aluminium bench that had previously been cleaned with alcohol to remove any olfactory cues. All treatments and both sexes were tested each day of test in order to prevent any impact of date on the results.

### Assessment of the level of choosiness

The level of choosiness of *H*. *affinis* was examined in test arenas under the 4 different experimental treatment levels of risk. We evaluated, at the individual carabid level, choosiness for a weed seed food item. Individual level of choosiness was assessed in “no choice” tests in which only one food type is offered to individuals [49]. Since most resources are encountered sequentially, animals cannot easily make comparative choices. Hence, no choice tests have been described as more ecologically realistic experimental designs [50–52]. No-choice tests have been proven useful and relevant in many studies [49,53–55] and are considered to be particularly suitable for measuring choosiness since an individual offered only one seed, and rejecting it, would be considered choosier than an individual accepting the seed [56–58]. In tests with multiple choices, alternative resources might impact on the choices an individual makes toward other resources, potentially leading to false negatives or positives [28,59]. Moreover, no choice tests are easier to standardize than multiple choice tests, which require that the focal individual has the sensory capability and the cognitive skills to compare several items simultaneously [49,59,60]. Lastly, longer latencies to acceptance of a food item, when there are no other simultaneously available options, might be interpreted as evidence for higher levels of choosiness [59].

**Fig 2:**
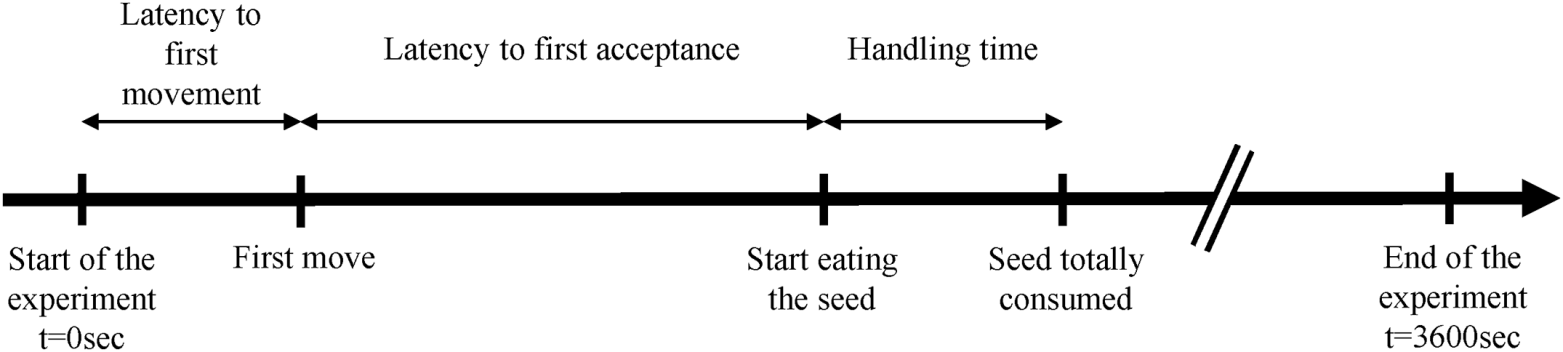
Schematic diagram of the chronological course of the experiment and the temporal metrics use in the tests. Latency to first movement was measured from the release of the test carabid to its first movement greater than its average body length; the latency to first acceptance of a seed was measured as the time from the first movement of an individual until it accepted the first seed; handling time is the duration of the seed consumption starting from an individual seizing the seed in its mandibles until it released the empty tegument. The experiment ended after a duration of 3600 s.

The level of choosiness was assessed by scoring four behaviours (Fig 2): i) the latency to the first movement of an individual (i.e. motion of more than the average body length); ii) the latency to first acceptance of a seed (i.e. from the first movement of an individual until it actually accept its first seed); iii) the handling time (i.e. the duration of the seeds consumptions) and, iv) the number of seeds eaten per individual during the 1 hour test. Given that the total number of seeds eaten might hide variation in behaviour in the test population, the proportion of individuals eating at least one seed during the test was also used in the analysis.

### Trajectometry

The presence of predators is expected to induce predator avoidance behaviours, such as reduced exploration or increased velocity. It is to be expected that such a change in locomotion or space use would reduce the probability of seed encounter and consequently the number of seeds eaten, irrespective of an individual’s level of choosiness. The trajectometry of each individuals was recorded during the one-hour test using a monochrome camera (IMAGINGSOURCE–model: DMK 31AU03) suspended above the arenas and connected to a computer. The video files of 29 individuals, from all treatments were lost due to a hard disk failure. The trajectometry data (n = 261 individuals: n = 66 for the control, n = 69 for the intraspecific competition, n = 63 for the interspecific competition and n = 63 for the predation treatments) were analysed using Ethovision (Noldus Information Technology, Wageningen, The Netherlands).

Differences in exploration behaviours were analysed as a function of the treatment. The tendency to stay in physical contact with borders of the arena (thigmotaxis) and to avoid open space (centrophobicity) were also assessed as proxies of an individual’s anxiety levels [61,62] and were expected to vary with the presence of predator cues. We therefore defined three annular zones corresponding to the “central zone” (0–5 cm), “inter-zone” (5–8 cm) and the “border zone” (8–9 cm) regions of the arena, delimited by the seed circles described above (Fig 1). The cumulative time spent within the central zone was scored as a measure of thigmotaxis and centrophobicity. The experimental area was divided up into 1 cm x 1 cm squares. We estimated the proportion of space used by scoring the number of squares visited at least once, by the focal carabid, as a proportion of the total number of squares (mean total number of squares per arena = 332.5, 95% CI = [331.4; 333.7]). Finally, mean velocity was scored as a proxy measure of activity, calculated by dividing the total distance travelled (cm) by the cumulative amount of time during which individuals were in movement (s).

### Statistical analysis

The data were analysed in R version 3.3.2 [63] (S1 Appendix). The number of seeds eaten per individual during the 1 hour test was modelled as a generalized linear model, assuming a negative binomial distribution. Because zero-inflated negative binomial model fitted the data better than the negative binomial model (Vuong’s test for non-nested models: p = 8.4×10^−5^, AIC = 37.9), we used ‘zeroinfl’ function from the ‘pscl’ package [64]. The proportion of individuals eating at least one seed during the test was analysed in each of the four different treatment levels using generalized linear modelling and binomial errors. The times of latency to first movement and first seed acceptance, and handling were analysed by means of the Cox proportional hazard models [65] in the ‘cox.ph’ function from the package ‘survival’ [66]. The Cox model allowed the analysis of censored data produced when a replicate was terminated before the end of the observed behaviour. For each Cox regression model fit, the proportional hazards assumption was assessed using the ‘cox.zph’ function. The velocity data and the cumulative time spent within the central zone were analysed using ANOVA. The data for the proportion of space used was arcsine transformed in order to meet the condition of normality for ANOVA.

For all parametric analyses, the full model included as effects the treatment level (control, intraspecific competition, interspecific competition and predation), the sex of the focal individual and their interactions. Significant effects of sex, treatment and their interactions were identified by sequential comparison of the nested sub-models, with and without a given covariate, using backward, stepwise elimination of non-significant variables and interaction terms. Where a global effect of treatments was detected, a post-hoc contrast analysis was performed.

To facilitate future meta-analysis or comparisons, we also reported effect size indices and the corresponding 95% confidence intervals [67,68]. When comparing means with non-normal data we used Cliff’s delta [69,70]. The measure of effect size for the latencies was the hazard ratio, estimated as the exponent of the regression coefficient, exp(beta), of the Cox model [71]. The hazard ratio was calculated either for the sex effect or the treatment effect. A sex hazard ratio above one indicates that the females had a longer latency time than that of the males. A treatment hazard ratio above 1 indicates that the treatment decreased the latency time compared to the control.

### Ethical note

This work followed the ABS/ASAB guidelines for the treatment of animals in behavioural research. Information about individuals’ origin, and housing conditions are described below. Transport between sampling site and laboratory, housing conditions, as well as monitoring of experimental arena, were done to reduce stress and maximise animal welfare.

## Results

### Latency to first movement

The latency to the first movement of an individual differed significantly between treatments (Cox model, χ^2^_3_ = 17.1, P < 0.001, S1 Fig). Compared to the control, it increased in the predation and intraspecific competition treatments but not in the interspecific treatment (Table 1). It was affected neither by the sex of the individual (Cox model, χ^2^_1_ = 0.25, P = 0.62, hazard ratio = 1.06, 95%CI = [0.74; 1.19]) nor the interaction between sex and treatment (Cox model, χ^2^_3_ = 3.40, P = 0.34).

**Table 1:** Contrast analysis between treatments for the latency to first movement.

	P	Hazard ratio	95%CI
Control—Predation	0.024	0.68	[0.49; 0.95]
Control—Intraspecific competition	0.0033	0.60	[0.43; 0.84]
Control—Interspecific competition	0.61	1.09	[0.79; 1.51]
Intraspecific competition—interspecific competition	< 0.001	1.82	[1.30; 2.54]
Intraspecific competition—predation	0.44	1.14	[0.82; 1.58]
Interspecific competition—predation	0.0052	0.63	[0.45; 0.87]

### Latency to first acceptance of a seed

**Fig 3:**
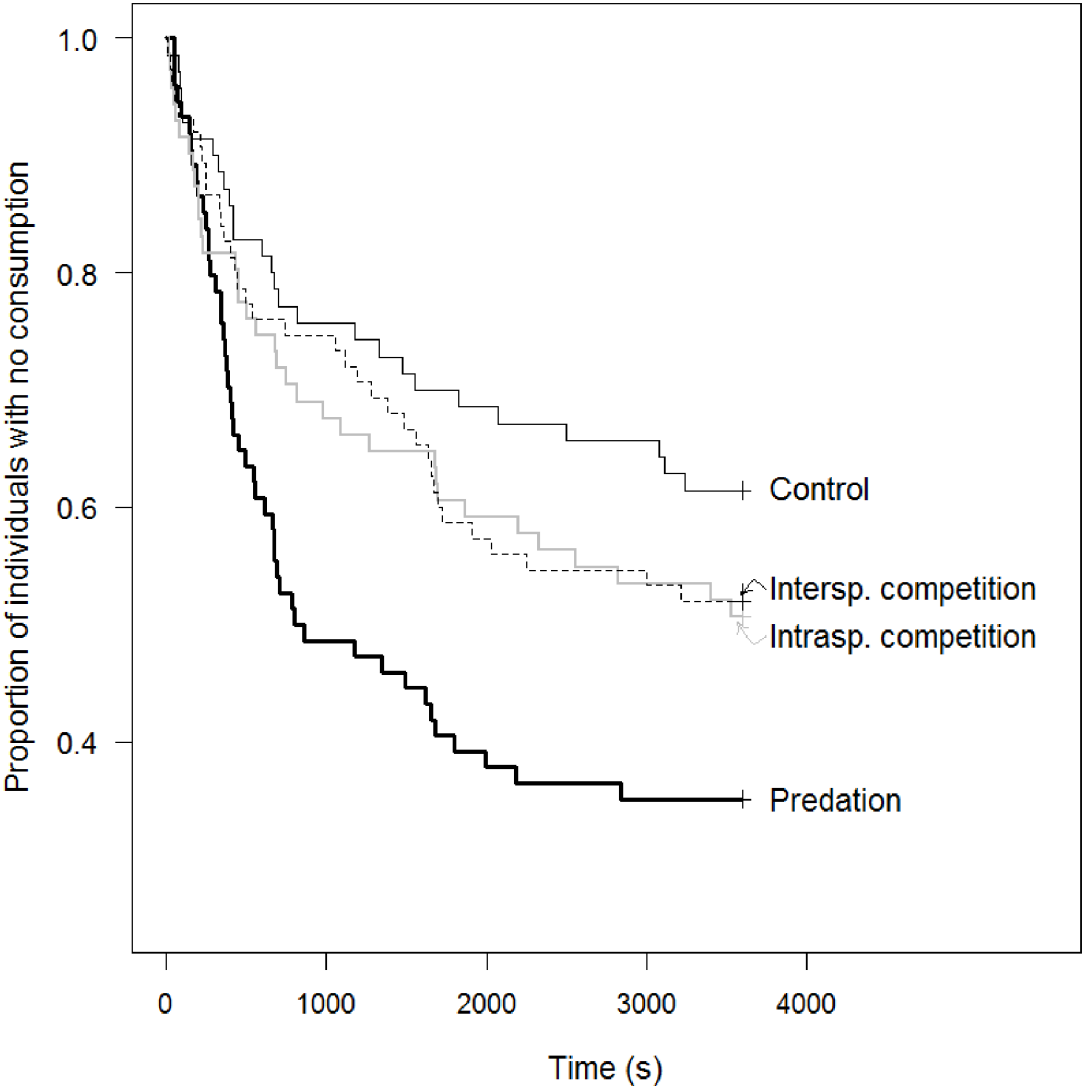
Kaplan-Meier plot for the latency to first acceptance as a function of the treatments. Each curve represents, for a given treatment level, the proportion of individuals with no consumption as a function of the time since the first move: control (continuous line, n = 70), intraspecific competition (grey line, n = 71), interspecific competition (dotted line, n = 75) and predation (bold line, n = 74). Individuals not eating before the end of the observation at time t = 3600 s were treated as censored data in the model.

The latency to first acceptance of a seed significantly differed between treatments (Fig 3, Cox model, χ^2^_3_ = 12.1, P = 0.007). The latency to first acceptance of a seed was shorter in the predation treatment than in all the three others treatments (Table 2).

**Table 2:** Contrast analysis between treatments for the latency to first acceptance of a seed.

	P	Hazard ratio	95%CI
Control—Predation	< 0.001	2.22	[1.38; 3.56]
Control—Intraspecific competition	0.19	1.39	[0.84; 2.30]
Control—Interspecific competition	0.26	1.33	[0.81; 2.19]
Intraspecific competition—interspecific competition	0.84	1.05	[0.66; 1.67]
Intraspecific competition—predation	0.04	1.59	[1.03; 2.46]
Interspecific competition—predation	0.02	1.67	[1.08; 2.57]

While non-significant, the values of effect size suggested that the latency to first acceptance of a seed was consistently shorter under the interspecific competition and intraspecific competition treatment than under the control treatment (Table 2). An *a posteriori* power analysis showed that such trends would have required a doubling of the sample size to become significant, provided that the mean value of effect size does not change. The latency to first acceptance of a seed did not differ between the two competition treatments (Table 2). It was also not affected by sex (Cox model, χ^2^_1_ = 2.22, P = 0.14, hazard ratio = 1.28, 95%CI = [0.92; 1.78]) or the interaction between sex and treatment (Cox model, χ^2^_3_ = 0.60, P = 0.90).

### Number of seeds eaten per individual and handling time

**Fig 4:**
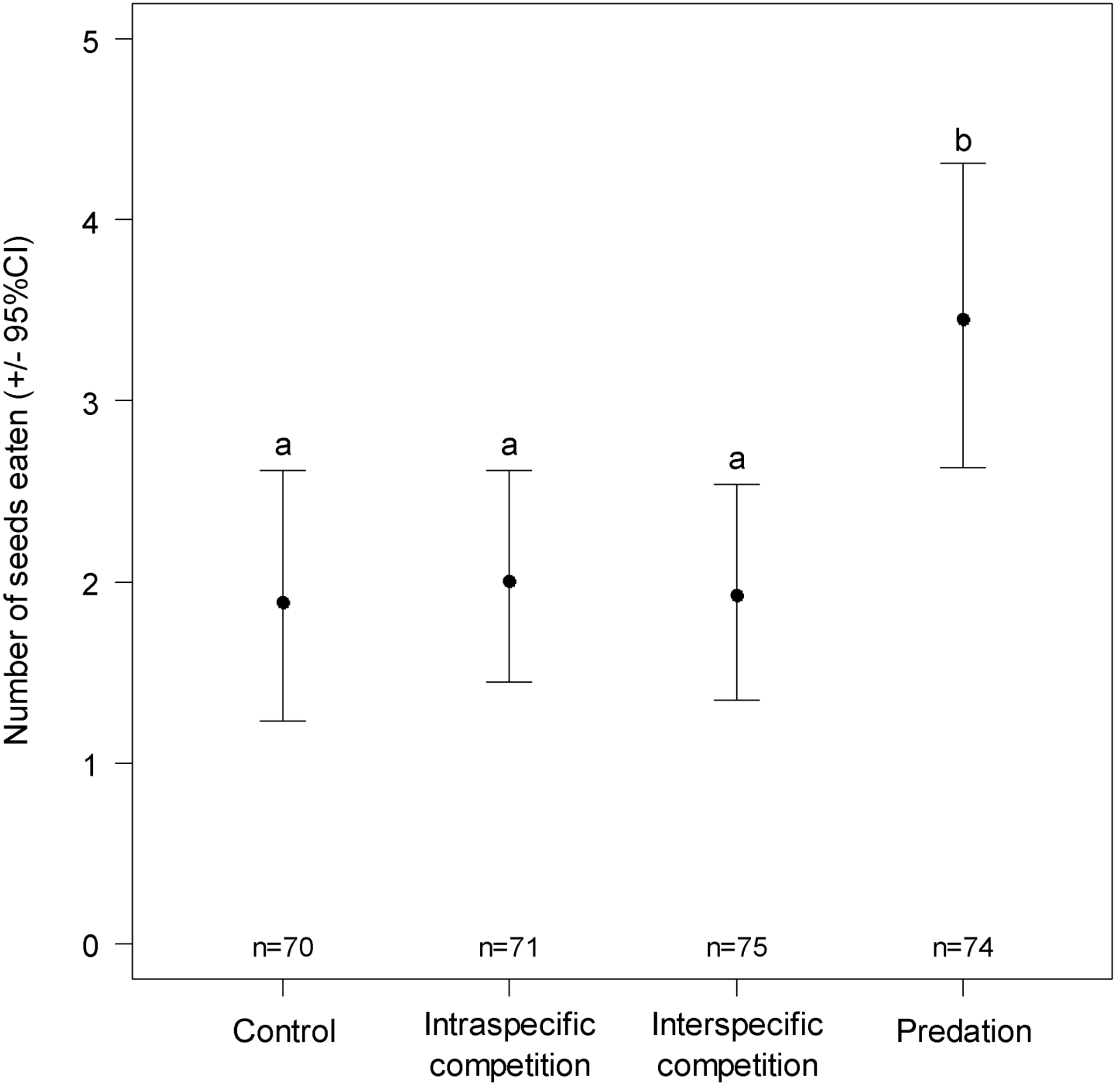
Mean number (bootstrapped +/- 95%CI) of seeds eaten per individuals after one hour of test in each treatment. Different letters correspond to statistically significant difference between treatments (post-hoc pairwise comparison with Tukey adjustment for multiple comparisons). The sample sizes are shown above the x-axis.

There was no significant effect of treatment on the handling time (Cox model, χ^2^_3_ = 1.9, P = 0.59, S2 Fig). There was a significant effect of the treatment on the mean number of seeds eaten per individual during the one hour test (Generalized linear model, χ^2^_6_ = 17.22, P = 0.009). The mean number of seeds eaten was significantly higher under the predation treatment than in the other treatments (Fig 4, Cliff’s delta for the difference between control and predation δ = 0.28, 95%CI = [0.10; 0.44]).

There was no statistical difference among the control and competition treatments. There was a significant effect of sex (Generalized linear model, χ^2^_2_ = 6.58, P = 0.037), but no sex by treatment interaction term (Generalized linear model, χ^2^_6_ = 1.47, P = 0.96). Females consumed more seeds than males (Cliff’s delta δ = 0.11, 95%CI = [0.01; 0.24], S3 Fig), with females having a mean consumption of 2.8 seeds (95%CI = [2.22; 3.43]) and males consuming 1.95 seeds (95%CI = [1.55; 2.39]) over the hour of testing.

The proportion of individuals that ate at least one seed in the hour of the test also differed between the treatments (Generalized linear model, χ^2^_3_ = 10.45, P = 0.015). Post-hoc comparisons with the control treatment showed that this proportion was significantly higher under the risk cues of predation (P = 0.003, odds-ratio = 2.94, 95%CI = [1.49; 5.79]), but not under the risk cues of intraspecific (P = 0.31, odds-ratio = 1.47, 95%CI = [0.76; 2.85]) or interspecific competition (P = 0.24, odds-ratio = 1.54, 95%CI = [0.79; 3.02]). There was no significant effect of sex (Generalized linear model, χ^2^_1_ = 1.35, P = 0.25) and no interaction between the sex and treatment effects (Generalized linear model, χ^2^_3_ = 0.23, P = 0.97).

### Trajectometry

**Fig 5:**
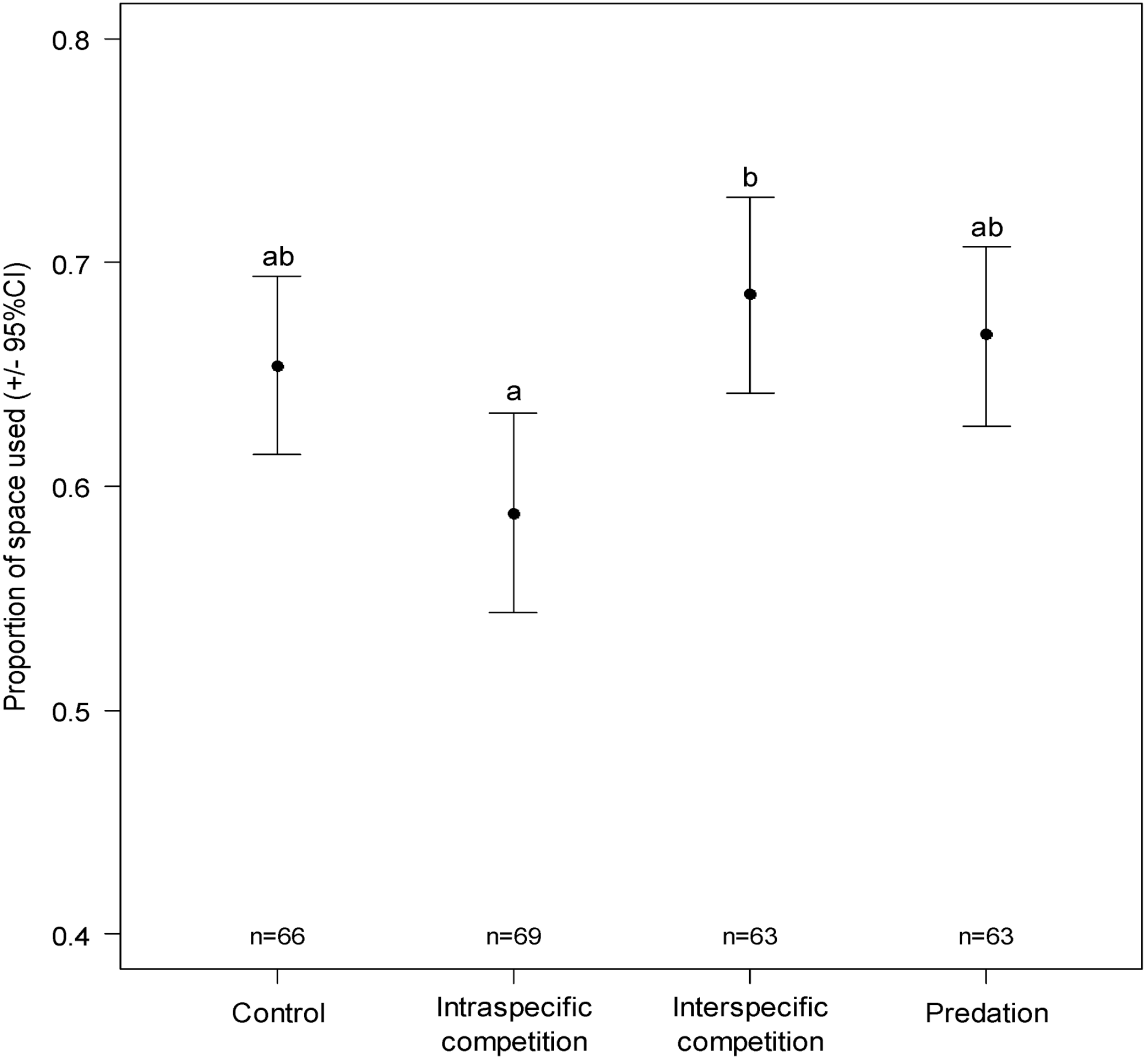
Proportion of space used (bootstrapped +/- 95%CI) after one hour of test in each treatment. Different letters correspond to statistically significant differences between treatments (post-hoc pairwise comparison with Tukey adjustment for multiple comparisons). The sample sizes are shown above the x-axis.

All individuals moved during the test. The mean velocity was not affected by the treatment (ANOVA, F_3, 257 =_ 0.36, P = 0.78), sex (ANOVA, F_1, 259_ = 1.51, P = 0.22) or the interaction between treatment and sex (ANOVA, F_253, 256_ = 1.74, P = 0.16). The proportion of space used differed between the two types of competition (ANOVA, F_3, 257_ = 3.36, P = 0.019), but did not differ between the predation treatment and the control (Fig 5). The cumulative time spent in the central zone was not affected either by treatment (ANOVA, F_3, 257_ = 1.58, P = 0.19), sex (ANOVA, F_1, 259_ = 0.034, P = 0.85) or the interaction between treatment and sex (ANOVA, F_253, 256_ = 0.26, P = 0.86).

## Discussion

### Adjustment of the foraging effort in response to predation risk

In order to allocate more time and energy to predator avoidance behaviours when exposed to predation risk, individuals should postpone foraging task [17]. This decrease of the foraging effort should not straightforwardly be interpreted as a reduction of the number of items consumed. Indeed, our results show that considering the number of items consumed as the sole metric of the intensity of an individual’s response to a risk of predation could be misleading. Individuals of the granivore, *H*. *affinis*, when exposed to chemicals cues of a potential predator were found to significantly increase the total number of food items consumed in comparison to the control or the competition treatments. This increase in the number of food items consumed suggest that individuals *H*. *affinis* reduced their level of choosiness toward feeding items. *Harpalus affinis* showed a marked reduction of the latency to first acceptance of a seed in comparison to the control or competitions treatments, suggesting that the effort that an individual is willing to invest in the acquisition of a resource (i.e. choosiness) is reduced under predation. Such foraging patterns cannot be interpreted as a lack of behavioural adjustment to the risk of predation or be explained by differences in handling time or trajectometry, as there were no differences in the handling time or the trajectometry metrics between the treatments.

Reductions in individual levels of choosiness could lead to the consumption of prey items that would be rejected under control conditions, but it might also provide important benefits. It could allow a greater focus on predator avoidance, for example, by reducing the cognitive load attributable to food item selection [72]. Metcalfe et al. [8,73] found that salmon exposed to a fake predator reduced their level of choosiness for passing food pellets. Given that salmon use vision to acquire information both for predator vigilance and for assessing the quality of their prey they might accept a potential reduction in food quality in order to focus on vigilance. Bees were observed to lower their threshold of acceptance of flower quality when exposed to potential ambush predation by cryptic crab spiders in flowers. In doing so, the bees were able to minimize conflict between foraging and predator vigilance and the high energetic costs of foraging flights [4].

The performance of any two tasks that use similar sensory machinery, such as vision or chemoreception, can result in “dual task interference” [74,75]. Due to limitations of cognitive load either one of the tasks could be detrimental to the other, thus producing an “outcome conflict” [75,76]. Even where these two tasks could be performed simultaneously, this will be both energy and time consuming [4] and many taxa do not succeed in solving the conflicts of dual task interference. Birds [77] and humans [78] have been observed failing to divide their attention between two complex visual tasks [4], for example. Hence, one solution to managing the limited available cognitive load, and the potential associated extra costs, might be to apply a weighting to each task [74]. In the vigilance-foraging trade-off this would be expressed by a reduction in the weight assigned to the foraging task, as was observed for salmon and bees [4,8,73]. Such difficulties in making acute choices, while performing a high-load cognitive task, were reviewed by Block *et al*. [72], who noted that individuals typically respond by reducing their period of judgment and making more rapid choices. Rodents living in patches without refugia have been shown to reduce their time exposed to predators by reducing the time spent choosing seed food items [9], lowering both the risk of starvation and the risk of predation [18,75].

Reductions in levels of choosiness for food items, as found for *H*. *affinis*, might therefore serve as a sensible strategy to reduce both the total duration of a foraging task and the cognitive load of the food quality assessment [7]. Our results therefore serve to extend the predation risk allocation hypothesis [18], by suggesting that individuals could adopt one of several alternative strategies, with both reductions and increases in their level of choosiness for food items being possible in risky situations. Future experimental assessments of the risk allocation hypothesis should, therefore, try to define “foraging effort” and take into account the process of decision making itself.

### Response to competition risk

A core expectation of our study was that the individuals should also decrease their level of choosiness in response to the risk of competition, due to opportunity costs [10]. We found that the effects of competition on the level of choosiness (i.e. latency to first acceptance and mean number of seeds eaten) were similar across the two competition treatments. Latency to first acceptance of a seed and mean number of seed eaten per individuals were also not significantly different between the competition treatments and to the control. However, the values of the effect size for the latencies to first acceptance would suggest at a reduction in individual levels of choosiness and that it would be misleading to interpret these results as evidence for absence of a competition effect [68]. Rather, it suggests that we may not have taken into account all possible co-variates of competition that affect foraging, such as individual personality [79,80], and future studies should seek to evaluate the importance of these co-variates.

In order to avoid agonistic behaviours or competitive interference between individuals, our protocol was based on indirect competition or predation risks in the form of olfactory cues impregnating the arena paper. It may be that the use of odour as a competition cue, in place of test competitor individuals and the associated reduction in food items that would have ensued, might have lowered the perceived risk of competition enough that the *H*. *affinis* individuals did not modify their foraging effort, irrespective of the potential linked costs [81]. Moreover, given that individuals were maintained in groups of up to 20 individuals prior to experiment, which matched the amount of individuals used to impregnate the tests papers, the focal individuals might have become habituated to situations of competition similar to the one under test potentially reducing our power to test for competition risk perception [82,83].

While changes in level of choosiness were not observed under both competition treatments, our results did demonstrate a difference in latency to first movement and in space use between the two competition treatments. *H*. *affinis* individuals were found to move later and visit fewer squares of the arena in the intraspecific competition treatment. Similar patterns in the use of space were observed for *P*. *melanarius* in avoiding papers impregnated with chemical cues from conspecifics [46]. We hypothesise that this lower space use and increased latency to first movement may be due to an effect of sex, with male and female arresting in the presence of odours from the opposite sex. An alternative hypothesis is that the perceived risk of competition itself affects space use. For example, individuals of the Bullethead Parrotfish, *Chlorurus spilurus*, do not change their feeding rate under competition, but modify the way that they use space during foraging [84].

### Carabid beetles in agroecosystem

Our study group of choice is the carabid beetles that naturally inhabit arable farmland. Many thousands of individuals exist in farm fields in communities of granivore, omnivore and predatory species that can be cannibalistic and inter-specific predators [36, Alice Charalabidis, pers. obs.]. Reductions in the level of choosiness, in an environment filled with predation cues, might lead to an increase in the number of weed seeds accepted by the granivorous carabids. Counterintuitively, therefore, predation risk might be a mechanism for a biodiversity-ecosystem function [85,86] relationship amongst the carabids. Rather than the commonly held expectation that communities formed of granivores alone should have the highest weed seed predation [87], our results predict that the ecological function of weed seed predation would increase with the diversity of the carabid community.

## Supporting information

S1 Fig Kaplan-Meier plot for the latency to first movement as a function of the treatments. Each curve represents, for a given treatment group, the proportion of individuals with no movement as a function of the time since the start of the experiment: control (continuous line, n = 70), intraspecific competition (grey line, n = 71), interspecific competition (dotted line, n = 75) and predation (bold line, n = 74). Individuals having not being observed moving before the end of the observation at time t = 3600 s were treated as censored data in the model.
(PDF)

S2 Fig Mean duration (bootstrapped +/- 95%CI) of handling time in each treatment. Different letters correspond to statistically significant difference between treatments (post-hoc pairwise comparison with Tukey adjustment for multiple comparisons). The sample sizes are shown above the x-axis.
(PDF)

S3 Fig Mean number (bootstrapped +/- 95%CI) of seeds eaten per individuals after one hour of test in each treatment separated by sex. Different letters correspond to statistically significant difference between treatments (post-hoc pairwise comparison with Tukey adjustment for multiple comparisons). The sample sizes are shown above the x-axis.
(PDF)

S1 Appendix Supporting dataset. All data needed to evaluate the conclusions in the paper are present in the paper and in the supplementary materials.
(ZIP)

## References

[1] Westneat DE, Fox CW, editors. Evolutionary behavioral ecology. Oxford: Oxford University Press; 2010 pp. 177–193.

[2] Chittka L, Osorio D. Cognitive dimensions of predator responses to imperfect mimicry. PLoS Biol. 2007;5: e339 doi: 10.1371/journal.pbio.0050339 18162048 10.1371/journal.pbio.0050339 PMC2222978

[3] Welti EAR, Putnam S, Joern A. Crab spiders (*Thomisidae*) attract insect flower-visitors without UV signalling. Ecol Entomol. 2016;41: 611–617. doi: 10.1111/een.12334

[4] Wang M-Y, Ings TC, Proulx MJ, Chittka L. Can bees simultaneously engage in adaptive foraging behaviour and attend to cryptic predators? Anim Behav. 2013;86: 859–866. doi: 10.1016/j.anbehav.2013.07.029

[5] Chittka L, Skorupski P, Raine NE. Speed–accuracy tradeoffs in animal decision making. Trends Ecol Evol. 2009;24: 400–407. doi: 10.1016/j.tree.2009.02.010 19409649 10.1016/j.tree.2009.02.010

[6] David M, Gillingham MAF, Salignon M, Laskowski KL, Giraldeau L-A. Speed–accuracy trade-off and its consequences in a scramble competition context. Anim Behav. 2014;90: 255–262. doi: 10.1016/j.anbehav.2014.02.009

[7] Leaver LA, Daly M. Effect of predation risk on selectivity in heteromyid rodents. Behav Processes. 2003;64: 71–75. doi: 10.1016/S0376-6357(03)00108-6 12914997 10.1016/s0376-6357(03)00108-6

[8] Metcalfe NB, Huntingford FA, Thorpe JE. Predation risk Impairs diet selection in juvenile salmon. Anim Behav. 1987;35: 931–933.

[9] Perea R, González R, San Miguel A, Gil L. Moonlight and shelter cause differential seed selection and removal by rodents. Anim Behav. 2011;82: 717–723. doi: 10.1016/j.anbehav.2011.07.001

[10] Dechaume-Moncharmont F-X, Brom T, Cézilly F. Opportunity costs resulting from scramble competition within the choosy sex severely impair mate choosiness. Anim Behav. 2016;114: 249–260. doi: 10.1016/j.anbehav.2016.02.019

[11] Underwood R. Vigilance behaviour in grazing african antelopes. Behaviour. 1982;79: 81–107. doi: 10.1163/156853982X00193

[12] Beauchamp G. What is the magnitude of the group-size effect on vigilance? Behav Ecol. 2008;19: 1361–1368. doi: 10.1093/beheco/arn096

[13] Sih A. Optimal behavior: can foragers balance two conflicting demands? Science. 1980;210: 1041–1043. doi: 10.1126/science.210.4473.1041 17797495 10.1126/science.210.4473.1041

[14] Milinski M, Heller R. Influence of a predator on the optimal foraging behaviour of sticklebacks (*Gasterosteus aculeatus L*.). Nature. 1978;275: 642–644. doi: 10.1038/275642a0

[15] Lima SL, Dill LM. Behavioral decisions made under the risk of predation: a review and prospectus. Can J Zool. 1990;68: 619–640. doi: 10.1139/z90-092

[16] Nonacs P, Blumstein DT. Predation risk and behavioral life history In: Westneat DF, Fox CW, editors. Evolutionary behavioral ecology. Oxford, UK: Oxford University Press; 2010.

[17] Higginson AD, Fawcett TW, Trimmer PC, McNamara JM, Houston AI. Generalized optimal risk allocation: foraging and antipredator behavior in a fluctuating Environment. Am Nat. 2012;180: 589–603. doi: 10.1086/667885 23070320 10.1086/667885

[18] Lima SL, Bednekoff P a. Temporal variation in danger drives antipredator behavior: the predation risk allocation hypothesis. Am Nat. 1999;153: 649–659. doi: 10.1086/303202 10.1086/303202 29585647

[19] Ferrari MCO, Sih A, Chivers DP. The paradox of risk allocation: a review and prospectus. Anim Behav. 2009;78: 579–585. doi: 10.1016/j.anbehav.2009.05.034

[20] Helfman GS. Threat-sensitive predator avoidance in damselfish-trumpetfish interactions. Behav Ecol Sociobiol. 1989;24: 47–58. doi: 10.1007/BF00300117

[21] Helfman GS, Winkelman DL. Threat sensitivity in bicolor damselfish: Effects of sociality and body size. Ethology. 1997;103: 369–383. doi: 10.1111/j.1439-0310.1997.tb00153.x

[22] Brown GE, Rive AC, Ferrari MCO, Chivers DP. The dynamic nature of antipredator behavior : prey fish integrate threat-sensitive antipredator responses within background levels of predation risk. Behav Ecol Sociobiol. 2006;61: 9–16. doi: 10.1007/s00265-006-0232-y

[23] Berger-tal O, Mukherjee S, Kotler BP, Brown JS. Complex state-dependent game between owls and gerbils. Ecol Lett. 2010;13: 302–310. doi: 10.1111/j.1461-0248.2010.01447.x 20455918 10.1111/j.1461-0248.2010.01447.x

[24] Sivy KJ, Ostoja SM, Schupp EW, Durham S. Effects of rodent species, seed species, and predator cues on seed fate. Acta Oecologica. 2011;37: 321–328. doi: 10.1016/j.actao.2011.03.004

[25] Godin J-GJ. Diet selection under the risk of predation Behavioural mechanisms of food selection. Berlin, Heidelberg: Springer Berlin Heidelberg; 1990 pp. 739–770. doi: 10.1007/978-3-642-75118-9_36

[26] Houtman R, Dill LM. The influence of predation risk on diet selectivity: A theoretical analysis. Evol Ecol. 1998;12: 251–261. doi: 10.1023/A:1006544031697

[27] Jennions MD, Petrie M. Variation in mate choice and mating preferences: a review of causes and consequences. Behav Ecol. 1997;72: 283–327. 10.1017/s0006323196005014 9155244

[28] Edward DA. The description of mate choice. Behav Ecol. 2014;0: 1–10. doi: 10.1093/beheco/aru142

[29] Lima SL, Valone TJ. Influence of predation risk on diet selection: a simple example in the grey squirrel. Anim Behav. 1986;34: 536–544. doi: 10.1016/S0003-3472(86)80122-1

[30] Ibrahim A. A., Huntingford FA. Laboratory and field studies of the effect of predation risk on foraging in Three-Spined Sticklebacks (*Gasterosteus aculeatus*). Behaviour. 1989;109: 46–57.

[31] Dianne L, Perrot-Minnot M-J, Bauer A, Guvenatam A, Rigaud T. Parasite-induced alteration of plastic response to predation threat: increased refuge use but lower food intake in *Gammarus pulex* infected with the acanothocephalan *Pomphorhynchus laevis*. Int J Parasitol. 2014;44: 211–216. doi: 10.1016/j.ijpara.2013.11.001 24291320 10.1016/j.ijpara.2013.11.001

[32] Davis JM, Nufio CR, Papaj DR. Resource quality or competition: why increase resource acceptance in the presence of conspecifics? Behav Ecol. 2011;22: 730–737. doi: 10.1093/beheco/arr042 22479135 10.1093/beheco/arr042 PMC3117901

[33] Amita H, Kawamori A, Matsushima T. Social influences of competition on impulsive choices in domestic chicks. Biol Lett. 2010;6: 183–186. doi: 10.1098/rsbl.2009.0748 19906684 10.1098/rsbl.2009.0748 PMC2865051

[34] McNamara JM, Houston AI. A general framework for understanding the effects of variability and interruptions on foraging behaviour. Acta Biotheor. 1987;36: 3–22. doi: 10.1007/BF00159228 3113143 10.1007/BF00159228

[35] Fox LR, Morrow PA. Specialization: species property or local phenomenon? Science. 1981;211: 887–893. doi: 10.1126/science.211.4485.887 17819016 10.1126/science.211.4485.887

[36] McKemey AR, Symondson WOC, Glen DM. Predation and prey size choice by the carabid beetle *Pterostichus melanarius* (Coleoptera: Carabidae): the dangers of extrapolating from laboratory to field. Bull Entomol Res. 2003;93: 227–234. doi: 10.1079/BER2003240 12762864 10.1079/BER2003240

[37] Foltan P. Influence of slug defence mechanisms on the prey preferences of the carabid predator *Pterostichus melanarius* (Coleoptera: Carabidae). Eur J Entomol. 2004;101: 359–364.

[38] Hatteland B, Grutle K, Mong CE, Skartveit J, Symondson WOC, Solhøy T. Predation by beetles (Carabidae, Staphylinidae) on eggs and juveniles of the Iberian slug *Arion lusitanicus* in the laboratory. Bull Entomol Res. 2010;100: 559–567. doi: 10.1017/S0007485309990629 20158927 10.1017/S0007485309990629

[39] Kromp B. Carabid beetles in sustainable agriculture: A review on pest control efficacy, cultivation impacts and enhancement. Agric Ecosyst Environ. 1999;74: 187–228. doi: 10.1016/S0167-8809(99)00037-7

[40] Currie CR, Spence JR, Niemelä J. Competition, cannibalism and intraguild predation among ground beetles (Coleoptera: Carabidae): A laboratory study. Coleopt Bull. 1996;50: 135–148.

[41] Petit S, Boursault A, Bohan DA. Weed seed choice by carabid beetles (Coleoptera: Carabidae): Linking field measurements with laboratory diet assessments. Eur J Entomol. 2014;111: 615–620. doi: 10.14411/eje.2014.086

[42] Honek A, Martinkova Z, Saska P, Pekar S. Size and taxonomic constraints determine the seed preferences of Carabidae (Coleoptera). Basic Appl Ecol. 2007;8: 343–353. doi: 10.1016/j.baae.2006.07.002

[43] Hammerstein P, Stevens JR. Six reasons for invoking evolution in decision theory Evolution and the mechanisms of decision making. The MIT Press; 2012 pp. 1–18. doi: 10.7551/mitpress/9780262018081.003.0001

[44] Law JJ, Gallagher RS. The role of imbibition on seed selection by *Harpalus pensylvanicus*. Appl Soil Ecol. 2015;87: 118–124. doi: 10.1016/j.apsoil.2014.11.015

[45] Lindroth CH. Coleoptera Carabidae Handbooks for the indentification of british insects. Royal Entomological Society; 1974 p. 98.

[46] Guy AG, Bohan DA, Powers SJ, Reynolds AM. Avoidance of conspecific odour by carabid beetles: a mechanism for the emergence of scale-free searching patterns. Anim Behav. 2008;76: 585–591. doi: 10.1016/j.anbehav.2008.04.004

[47] Armsworth CG. The influence of a carabid beetle predator on the behaviour and dispersal of slug pests. PhD Thesis. Cardiff University. 2005.

[48] Armsworth CG, Bohan DA, Powers SJ, Glen DM, Symondson WOC. Behavioural responses by slugs to chemicalsfrom a generalist predator. Anim Behav. 2005;69: 805–811. doi: 10.1016/j.anbehav.2004.07.009

[49] Dougherty LR, Shuker DM. Precopulatory sexual selection in the seed bug *Lygaeus equestris*: a comparison of choice and no-choice paradigms. Anim Behav. 2014;89: 207–214. doi: 10.1016/j.anbehav.2014.01.005

[50] Allison JD, Cardé RT. Male pheromone blend preference function measured in choice and no-choice wind tunnel trials with almond moths, *Cadra cautella*. Anim Behav. 2008;75: 259–266. doi: 10.1016/j.anbehav.2007.04.033

[51] Larrinaga AR. A univariate analysis of variance design for multiple-choice feeding-preference experiments: A hypothetical example with fruit-eating birds. Acta Oecologica. 2010;36: 141–148. doi: 10.1016/j.actao.2009.11.003

[52] Rapport DJ, Turner JE. Determination of predator food preferences. J Theor Biol. 1970;26: 365–372. doi: 10.1016/0022-5193(70)90089-5 5462334 10.1016/0022-5193(70)90089-5

[53] Wagner WE. Measuring female mating preferences. Anim Behav. 1998;55: 1029–1042. doi: 10.1006/anbe.1997.0635 9632487 10.1006/anbe.1997.0635

[54] Dougherty LR, Shuker DM. The effect of experimental design on the measurement of mate choice: a meta-analysis. Behav Ecol. 2015;26: 311–319. doi: 10.1093/beheco/aru125

[55] Martel V, Boivin G. Do choice tests really test choice? J Insect Behav. 2011;24: 329–336. doi: 10.1007/s10905-011-9257-9

[56] Reinhold K, Schielzeth H. Choosiness, a neglected aspect of preference functions: a review of methods, challenges and statistical approaches. J Comp Physiol A. 2015;201: 171–182. doi: 10.1007/s00359-014-0963-6 25398575 10.1007/s00359-014-0963-6

[57] Rodriguez RL, Greenfield MD, Rodríguez RL, Greenfield MD, Rodriguez RL, Greenfield MD, et al Genetic variance and phenotypic plasticity in a component of female mate choice in an ultrasonic moth. Evolution. 2003;57: 1304–1313. http://dx.doi.org/10.1554/02-446 12894938 10.1111/j.0014-3820.2003.tb00338.x

[58] Rothbart MM, Hennig RM. Calling song signals and temporal preference functions in the cricket *Teleogryllus leo*. J Comp Physiol A. 2012;198: 817–825. doi: 10.1007/s00359-012-0751-0 22945775 10.1007/s00359-012-0751-0

[59] Murray TJ, Withers TM, Mansfield S. Choice versus no-choice test interpretation and the role of biology and behavior in parasitoid host specificity tests. Biol Control. 2010;52: 153–159. doi: 10.1016/j.biocontrol.2009.10.003

[60] Raffa KF, Havill NP, Nordheim E V. How many choices can your test animal compare effectively? Evaluating a critical assumption of behavioral preference tests. Oecologia. 2002;133: 422–429. doi: 10.1007/s00442-002-1050-1 28466207 10.1007/s00442-002-1050-1

[61] Tremmel M, Muller C. Insect personality depends on environmental conditions. Behav Ecol. 2013;24: 386–392. doi: 10.1093/beheco/ars175

[62] Soibam B, Mann M, Liu L, Tran J, Lobaina M, Kang YY, et al Open-field arena boundary is a primary object of exploration for Drosophila. Brain Behav. 2012;2: 97–108. doi: 10.1002/brb3.36 22574279 10.1002/brb3.36 PMC3345355

[63] R Development Core Team, (2016) R: A language and environment for statistical computing. R Foundation for Statistical Computing Vienna, Austria http://www.R-project.org

[64] Jackman Simon (2015). pscl: Classes and methods for R developed in the political science computational laboratory, Stanford University. Department of political science, Stanford University. Stanford, California. R package version 1.4.9. URL: http://pscl.stanford.edu/

[65] Dechaume-Moncharmont F-X, Azzouz H, Pons O, Pham-Delègue M-H. Soybean proteinase inhibitor and the foraging strategy of free flying honeybees. Apidologie. 2005;36: 421–430. doi: 10.1051/apido:2005031

[66] Therneau T (2006) A package for survival analysis, version 2.38, <URL: http://CRAN.R-project.org/package=survival>

[67] Lakens D. Calculating and reporting effect sizes to facilitate cumulative science: a practical primer for t-tests and ANOVAs. Front Psychol. 2013;4: 863 doi: 10.3389/fpsyg.2013.00863 24324449 10.3389/fpsyg.2013.00863 PMC3840331

[68] Nakagawa S, Cuthill IC. Effect size, confidence interval and statistical significance: a practical guide for biologists. Biol Rev. 2007;82: 591–605. doi: 10.1111/j.1469-185X.2007.00027.x 17944619 10.1111/j.1469-185X.2007.00027.x

[69] Cliff N, Keats JA. Ordinal measurement in the behavioral Sciences Psychology. New york, USA; 2003.

[70] Monceau K, Moreau J, Richet J, Motreuil S, Moret Y, Dechaume-Moncharmont F. Larval personality does not predict adult personality in a holometabolous insect. Biol J Linn Soc. 2017;120: 869–878. doi: 10.1093/biolinnean/blw015

[71] Dechaume-Moncharmont F-X, Decourtye A, Hennequet-Hantier C, Pons O, Pham-Delègue M-H. Statistical analysis of honeybee survival after chronic exposure to insecticides. Environ Toxicol Chem. 2003;22: 3088–3094. doi: 10.1897/02-578 14713054 10.1897/02-578

[72] Block RA, Hancock PA, Zakay D. How cognitive load affects duration judgments: A meta-analytic review. Acta Psychol. 2010;134: 330–343. doi: 10.1016/j.actpsy.2010.03.006 20403583 10.1016/j.actpsy.2010.03.006

[73] Metcalfe NB, Huntingford FA, Thorpe JE. The influence of predation risk on the feeding motivation and foraging strategy of juvenile Atlantic salmon. Anim Behav. 1987;35: 901–911. doi: 10.1016/S0003-3472(87)80125-2

[74] Pashler H. Dual-task interference in simple tasks : Data and Theory. Psychol Bull. 1994;116: 220–244. 7972591 10.1037/0033-2909.116.2.220

[75] Lawrence ES. Vigilance during “easy” and “difficult” foraging tasks. Anim Behav. 1985;33: 1373–1375. doi: 10.1016/S0003-3472(85)80206-2

[76] Navon D, Miller J. Role of outcome conflict in dual-task interference. J Exp Psychol Hum Percept Perform. 1987;13: 435–448. doi: 10.1037/0096-1523.13.3.435 2958592 10.1037//0096-1523.13.3.435

[77] Dukas R. The cost of limited attention in blue jays. Behav Ecol. 2000;11: 502–506. doi: 10.1093/beheco/11.5.502

[78] Joseph JS, Chun MM, Nakayama K. Attentional requirements in a “preattentive” feature search task. Nature. 1997;387: 805–807. doi: 10.1038/42940 9194560 10.1038/42940

[79] David M, Cézilly F, Giraldeau L-A. Personality affects zebra finch feeding success in a producer–scrounger game. Anim Behav. 2011;82: 61–67. doi: 10.1016/j.anbehav.2011.03.025

[80] Royauté R, Pruitt JN. Varying predator personalities generates contrasting prey communities in an agroecosystem. Ecology. 2015;96: 2902–2911. doi: 10.1890/14-2424.1.sm 27070010 10.1890/14-2424.1

[81] Mitchell W a, Abramsky Z, Kotler BP, Pinshow B, Joel S. The effect of competition on foraging activity in desert rodents : Theory and Experiments. Ecology. 1990;71: 844–854.

[82] Mohamad R, Wajnberg E, Monge J-P, Goubault M. The effect of direct interspecific competition on patch exploitation strategies in parasitoid wasps. Oecologia. 2014;177: 305–315. doi: 10.1007/s00442-014-3124-2 25367579 10.1007/s00442-014-3124-2

[83] Milinski M. Optimal foraging: The influence of intraspecific competition on diet selection. Behav Ecol Sociobiol. 1982;11: 109–115. doi: 10.1007/BF00300099

[84] Davis K, Carlson PM, Bradley D, Warner RR, Caselle JE. Predation risk influences feeding rates but competition structures space use for a common Pacific parrotfish. Oecologia. 2017;184: 139–149. doi: 10.1007/s00442-017-3857-9 28342012 10.1007/s00442-017-3857-9

[85] Hines J, van der Putten WH, De Deyn GB, Wagg C, Voigt W, Mulder C, et al Towards an integration of biodiversity–ecosystem functioning and food web theory to evaluate relationships between multiple ecosystem services Advances in Ecological Research. 1st ed Elsevier Ltd.; 2015 pp. 161–199. doi: 10.1016/bs.aecr.2015.09.001

[86] Reiss J, Bridle JR, Montoya JM, Woodward G. Emerging horizons in biodiversity and ecosystem functioning research. Trends Ecol Evol. 2009;24: 505–514. doi: 10.1016/j.tree.2009.03.018 19595476 10.1016/j.tree.2009.03.018

[87] Petit S, Bohan DA. The use of insects in integrated weed management In: Zimdahl Bob, editor. Integrated weed management for sustainable agriculture. Burleigh dodds Science publishing; 2017.

