# Supplementary material for: Risk of predation makes foragers less choosy about their food
Source: PLoS One. 2017 Nov 9;12(11):e0187167. doi: 10.1371/journal.pone.0187167 (PMC5679636; doi:10.1371/journal.pone.0187167)
Supplement: S1 Fig — Each curve represents, for a given treatment group, the proportion of individuals with no movement as a function of the time since the start of the experiment: control (continuous line, n = 70), intraspecific competition (grey line, n = 71), interspecific competition (dotted line, n = 75) and predation (bold line, n = 74). Individuals having not being observed moving before the end of the observation at time t = 3600 s were treated as censored data in the model. (PDF) [file pone.0187167.s001.pdf]

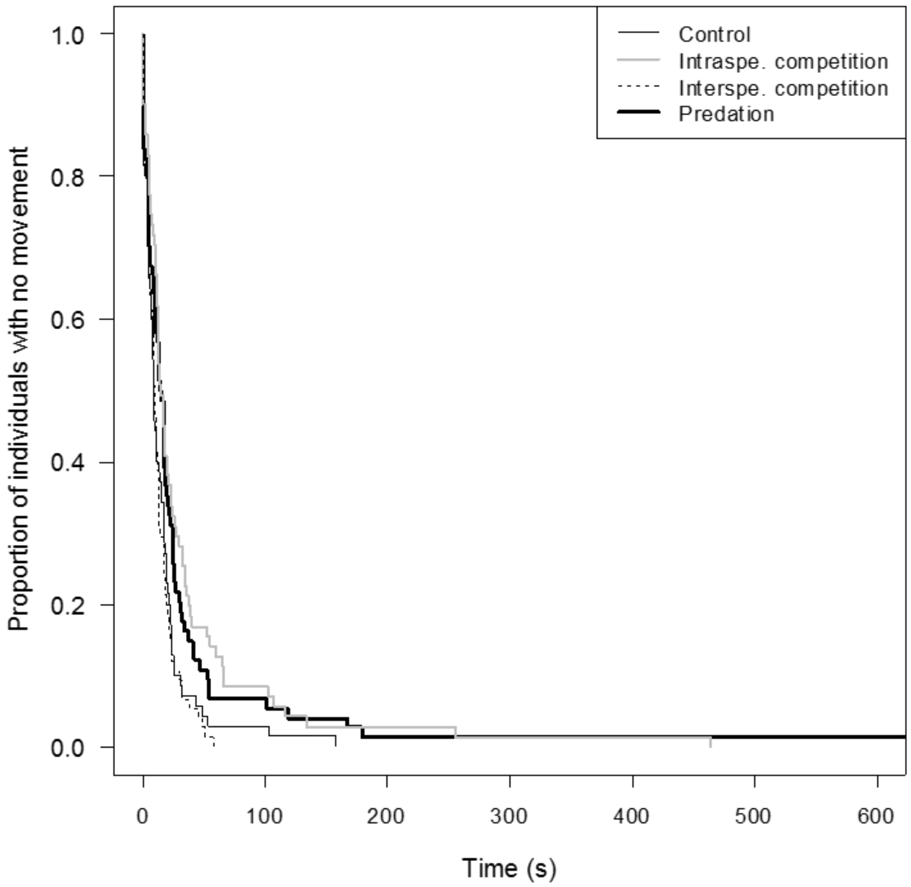

S1 Fig.: Kaplan-Meier plot for the latency to first movement as a function of the treatments. Each curve represents, for a given treatment group, the proportion of individuals with no movement as a function of the time since the start of the experiment: control (continuous line, n = 70), intraspecific competition (grey line, n = 71), interspecific competition (dotted line, n = 75) and predation (bold line, n = 74). Individuals having not being observed moving before the end of the observation at time t = 3600 s were treated as censored data in the model.
