# Supplementary material for: Risk of predation makes foragers less choosy about their food
Source: PLoS One. 2017 Nov 9;12(11):e0187167. doi: 10.1371/journal.pone.0187167 (PMC5679636; doi:10.1371/journal.pone.0187167)
Supplement: S3 Fig — Different letters correspond to statistically significant difference between treatments (post-hoc pairwise comparison with Tukey adjustment for multiple comparisons). The sample sizes are shown above the x-axis. (PDF) [file pone.0187167.s003.pdf]

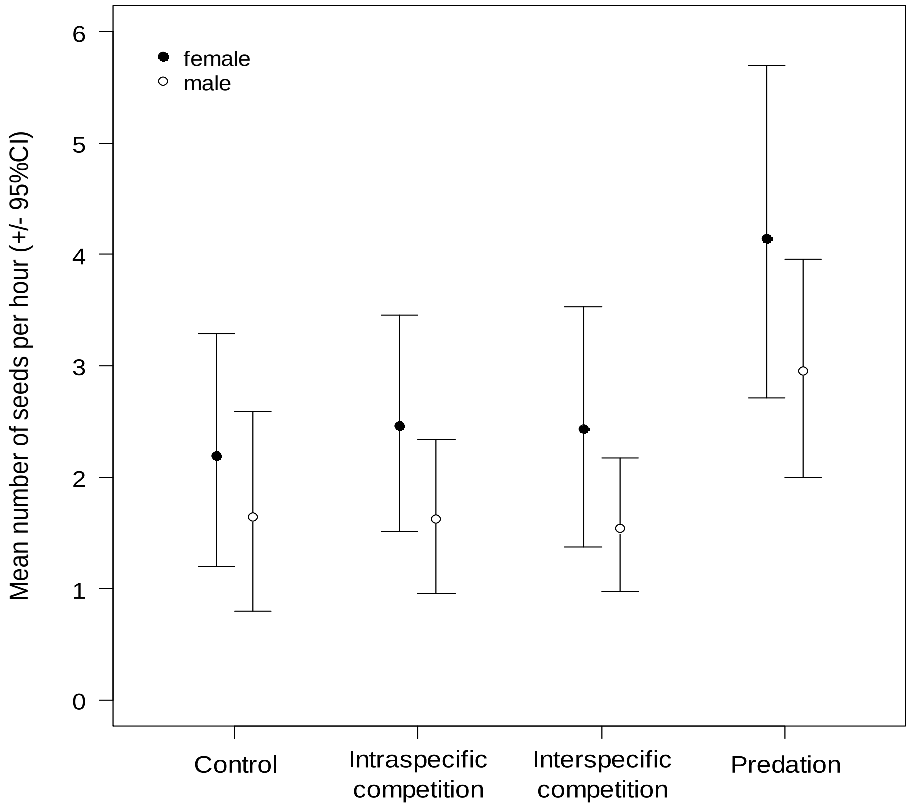

S3 Fig.: Mean number (bootstrapped +/- 95%CI) of seeds eaten per individuals after one hour of test in each treatment separated by sex. Different letters correspond to statistically significant difference between treatments (post-hoc pairwise comparison with Tukey adjustment for multiple comparisons). The sample sizes are shown above the x-axis.
